# The nuclear envelope and metastasis

**DOI:** 10.18632/oncotarget.28375

**Published:** 2023-04-14

**Authors:** Emily Hansen, James M. Holaska

**Keywords:** emerin, metastasis, mechanotransduction, breast cancer, nucleoskeleton

Nuclear morphology is one of the basic visual criteria used by pathologists to diagnose breast cancer. Immunofluorescence staining of the nuclear structural proteins lamin B and emerin was recommended as an effective diagnostic tool for both thyroid and breast cancer [1–3], suggesting nuclear structure is intimately tied to malignant transformation. But what role nuclear morphology plays in cancer transformation and progression remains unclear.

The most likely explanation for why cancer cells present with distinct nuclear morphology is thought to be related to the most likely route of cancer spread: the vasculature. For a tumor to metastasize, cancer cells need to enter and exit the blood and lymphatic vessels by squeezing through extremely small gaps in the endothelium, most of which are 1.2–2 μm in diameter [4]. While the cytoplasm is very flexible and the cytoskeleton can rearrange to fit through openings as narrow as 1 μm, the nuclear diameter (10–20 μm) and its considerable stiffness (2–10x stiffer than the cytoplasm) [5] represent physical barriers to this process. Thus, to enable metastasis, cancer cells must also increase their nuclear malleability.

Studies have shown that nuclear softening is associated with tumor aggressiveness and metastasis [6–8]. Although nuclear softening is one of the ‘hallmarks of cancer’ [9, 10] it remains poorly understood. Nuclear shape and stiffness are governed by a complex set of structural proteins that serve as both scaffolds and signaling proteins to influence almost all aspects of nuclear function. The best studied nucleostructural proteins are lamins, which are frequently downregulated in cancer [5, 11]. However, it is difficult to ascertain whether specific functional consequences are due to lamins or due to displacement of lamin-interacting proteins upon lamin loss. For example, nuclear size and shape is also governed by emerin [5, 11–13], which binds to lamins at the nuclear envelope (NE) and upon lamin loss is retained in the endoplasmic reticulum [14]. Like lamins, emerin is frequently mutated in cancer [15], with mutations in its transmembrane and actin-binding domains. We found that in breast cancer, emerin expression in tumor tissue is significantly correlated to survival time [16]. These data suggest emerin plays a central role in pathogenic transformation and progression of malignant breast tissue.

Despite the evidence linking nuclear shape and invasion, little effort has been devoted to understanding the mechanistic link between emerin deregulation and breast cancer progression. We evaluated emerin levels and nuclear shape in normal and breast cancer cell lines and found that triple-negative breast cancer cells have decreased emerin levels [16]. As expected, breast cancer cells also displayed smaller and more irregular nuclei. To understand if nuclear deformation in breast cancer was directly related to emerin, we expressed GFP-emerin in cells and found it increased nuclear area in cancer cells (MDA-231), but had no effect in normal breast epithelial cells (MCF10A) [16]. Recently, increased emerin expression was found to increase nuclear stiffness in single-nuclei studies of melanoma cells [17]. Meanwhile, depleting emerin was shown to decrease nuclear structure and stability and increase both invasion and migration [18].

Emerin is a ubiquitously expressed integral inner nuclear membrane protein, which in addition to nuclear structure, also influences genomic organization, cell signaling, and gene expression [19]. Therefore, it was unclear which of these functions was critical for blocking cancer cell invasion and metastasis. Emerin’s functions are directly related to its ability to physically interact with specific partners [20]. Several alanine-substitution mutants of emerin were created with specific consequences on emerin binding to proteins relevant to its cellular functions, including lamin A (for formation of the nuclear lamina) and actin (for nucleoskeletal structure). Introducing GFP-emerin rescued nuclear area, volume, and circularity while the negative control failed to do the same [16]. Introducing a GFP-emerin nucleoskeletal binding mutant that disrupted binding to lamins and actin was unable to rescue nuclear integrity, migration, and invasion [16], suggesting emerin binding to lamin A and/or actin is necessary for its ability to affect nuclear size and shape [16].

Transwell assays were used to test if rescuing nuclear structure was related to its ability to affect cell migration and invasion. Wild-type (wt) emerin decreased both migration and invasion in MDA-231 cells, but the nucleoskeletal binding mutant failed to do the same [16]. This effect was not caused by a general motility failure or the cells’ inability to respond to signaling cues, as cells expressing either wt emerin or emerin mutants demonstrated no differences in their ability to close scratch-wounds [16]. These findings showed that binding to the nucleoskeleton was critical for emerin’s ability to block metastatic properties *in vitro*.

To examine if the failure to migrate *in vitro* translated to failure to metastasize *in vivo*, we used an orthotopic mouse model of breast cancer. In addition to GFP-emerin, the MDA-231 cells also expressed infrared fluorescent protein, which allowed us to monitor them *in vivo*. We found that wt emerin decreased primary tumor size and tumor growth rate, an effect that was lost in emerin mutants with compromised binding to lamin A and actin [16]. Most relevant to the migratory function of breast cancer cells, wt emerin blocked the ability of MDA-231 cells to metastasize to the lung [16]. This effect was dependent on emerin’s ability to bind to lamin A and actin, as the nucleoskeletal binding mutant failed to replicate this phenomenon. This metastatic effect was not caused by reduced tumor burden [16], demonstrating that the function of emerin in metastatic spread is likely independent of its role in primary tumor growth. In agreement with these findings [16], Reis-Sobreiro et al. reported that loss of emerin led to instability of nuclear shape, increased migration and invasion, and to widespread metastasis of prostate cancer cells *in vivo* [18]. This suggests that loss of emerin may be a generalized mechanism for metastatic transformation to drive nuclear softening.

It is unclear what role emerin plays in nuclear softening in response to changes in the tumor microenvironment (TME). Nuclear and cellular stiffness are regulated by the stiffness of the TME, which is caused by increased extracellular matrix (ECM) secreted by the tumor [21, 22], with increased ECM stiffness associated with nuclear softening. This stiffening was shown to further trigger mechanotransduction events, many of which are related to migration and invasiveness [23]. Decreased emerin is seen upon increased ECM stiffening [24], suggesting that emerin plays a role in responding to changes in the TME. The linker of nucleoskeleton and cytoskeleton (LINC) complex is the major conduit for transducing mechanical signals (i.e., changes in ECM stiffness) from the cell surface to the nucleus. Emerin binds the LINC complex via SUN-domain proteins [25], and the LINC complex’s transmission of mechanical force to the nucleus causes emerin-dependent nuclear stiffness changes [26]. Additionally, in response to force, phosphorylation of emerin leads to nuclear stiffening [27]. However, we still do not understand how stiffness in the TME is transduced by the LINC complex and what role emerin plays in responding to this signal.

**Figure 1:**
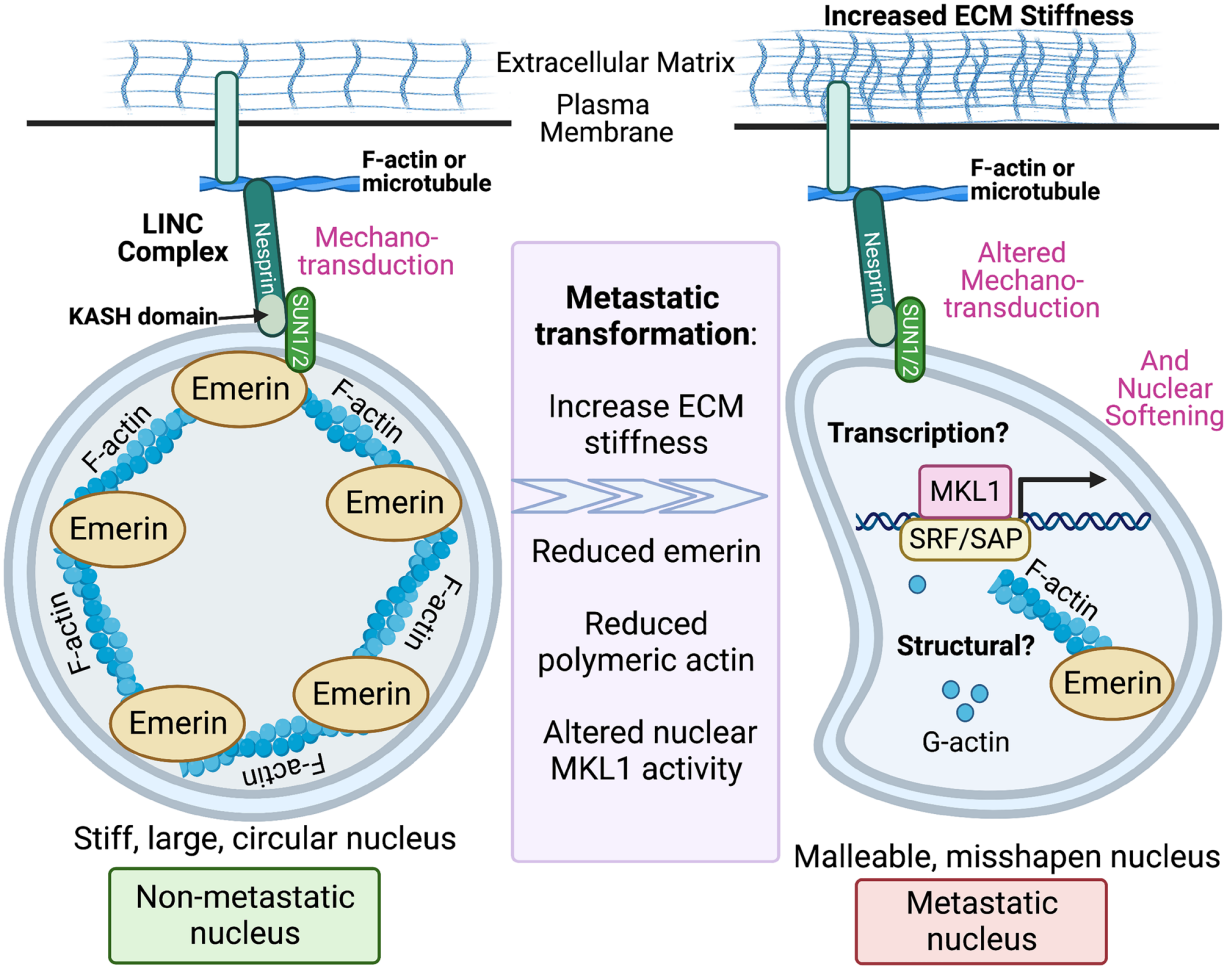
Working model for TME-mediated nuclear softening via LINC and emerin. Emerin binding to nuclear F-actin and the LINC complex maintains the structural integrity of the nucleus. Emerin is predicted to be a key effector of mechanotransduction to transduce mechanical signals to the nucleus to facilitate genetic and biochemical changes. Changes in TME is predicted to elicit transcriptional and/or structural responses through LINC and emerin to cause nuclear softening. Disruption of these interactions is predicted to enable metastatic transformation of cancer cells. Created with http://BioRender.com.

Previous work showed that changes in substrate stiffness cause LINC-dependent changes in nuclear filamentous actin (F-actin) and in the transcription of migratory and invasive genes by pro-oncogenic transcription factor megakaryoblastic leukemia protein-1 (MKL1) [26, 28, 29]. In response to substrate-stiffening, emerin was shown to be required for regulating substrate stiffness-dependent activation of the transcriptional co-activator complex SRF-MKL1, which is responsible for activating expression of genes involved in nuclear structure [26]. Because emerin is a modulator of actin polymerization [30] and MKL1 is regulated by nuclear actin dynamics [20, 31] we posit that emerin is a mechanosensor that responds to extracellular stimuli through the LINC complex by modulating structural and/or transcriptional outcomes, resulting in nuclear softening and driving metastasis in triple-negative breast cancer (Figure 1). However, it remains to be determined 1) whether it is the structural or transcriptional outputs that are driving progression; 2) how emerin transduces or senses these mechanical signals; and 3) how emerin reduction or mutation causes dysfunctional mechanotransduction in cancer.

We propose that emerin binding to F-actin and the LINC complex is required for the structural integrity of the nucleus – and that disruption of these interactions enables metastatic transformation of breast cancer cells through nuclear softening (Figure 1). Loss of emerin or its function is likely an early step in the metastatic cascade and provides a selective advantage for dissemination of metastatic cells. Thus, it is important to investigate how the interaction of emerin with the nucleoskeleton modulates downstream pathways, including transcriptional activation of pro-migratory proteins, to not only understand how emerin controls metastasis in breast cancer, but also to identify novel downstream pathways that could be druggable targets. Breast cancer metastasis remains the most lethal event in the disease course and the current lack of treatment options drives the negative patient outcomes. Therefore, ascertaining the mechanisms that enable metastatic spread can be transformational in our understanding of tumor progression and for future development of therapeutic interventions.
